# Suboptimal dengue genome leverages non-canonical translation mechanisms

**DOI:** 10.1016/j.isci.2025.112428

**Published:** 2025-04-15

**Authors:** Priyanka Mehta, Priti Devi, Sandeep Budhiraja, Bansidhar Tarai, Rajesh Pandey

**Affiliations:** 1 Division of Immunology and Infectious Disease Biology, INtegrative GENomics of HOst-PathogEn (INGEN-HOPE) Laboratory, CSIR-Institute of Genomics and Integrative Biology (CSIR-IGIB), Mall Road, Delhi 110007, India; 2 Academy of Scientific and Innovative Research (AcSIR), Ghaziabad 201002, India; 3 Max Super Speciality Hospital (A Unit of Devki Devi Foundation), Max Healthcare, Delhi 110017, India

**Keywords:** Microbiology, Genomics

## Abstract

Dengue is a notable example of vector-borne RNA virus responsible for severe hemorrhagic fever. Its compact genome necessitates reliance on the host’s translational machinery for replication. This study investigates the plausible adaptive strategies employed by dengue serotypes for effective translation within the human host. By analyzing viral reads from the RNA-seq dataset derived from the hospital-admitted patients, we explored the impact of dinucleotide diversity on codon optimization, and compatibility of serotypes with the host. Our findings reveal only moderate congruency of serotypes with the host and identified genomic composition common to several RNA viruses. Notably, unique coverage patterns were observed within the genome of DENV-2 serotypes. Using ribosome profileing (Ribo-seq) data, we extended our analysis to assess the translatability of potential internal open reading frames (iORFs) identified in the RNA-seq dataset. Nine common iORFs were identified across both the datasets, underscoring potential non-canonical translational mechanisms that might enhance DENV genome optimization.

## Highlights

• Dengue serotypes show gene-specific (di)nucleotide patterns
• Dengue shows a preference for A codons, with bias against codons ending in C or G
• DENV2 shows unique genome coverage, with regions aligning to small internal ORFs (iORFs)
• iORFs with translation support from independent Ribo-seq data

## Graphical abstract

**Figure:**
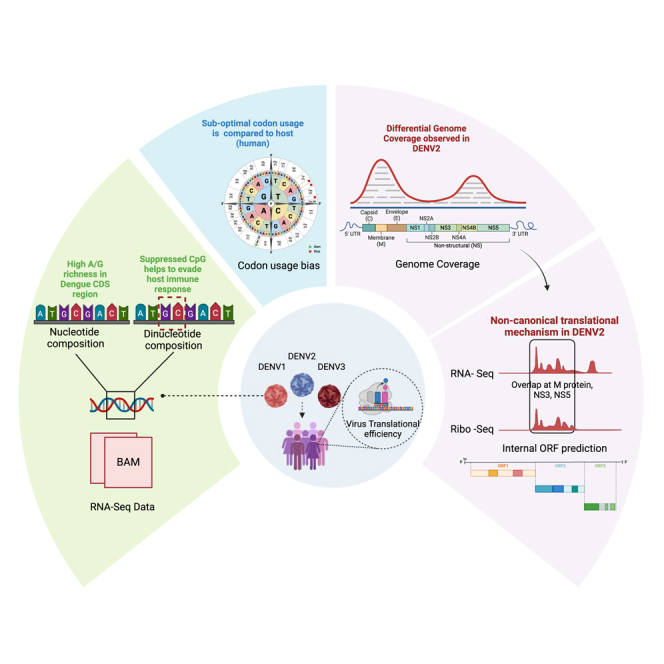

## Introduction

RNA viruses exhibit remarkable diversity in their structure and genome sizes, ranging from the lipid-rich envelopes characteristic of flaviviruses to the massive genomes as in coronaviruses (∼30 kb). These viruses are associated with a broad spectrum of human diseases ranging from mild upper respiratory tract infection to hemorrhagic fever.^1^ However, despite the genetic diversity, the general strategy employed to replicate the viral genome often follows a similar pattern. Most RNA viruses encode/carry the necessary enzymes for independent genome replication, but when it comes to protein synthesis, they are mostly dependent on the host’s translation machinery.^2^ RNA viruses intricately adopt the host’s cellular machinery to ensure efficient replication and survival.^3^ Upon entering the host cell, they rapidly initiate transcription and translation, often bypassing nuclear processes by utilizing cytoplasmic machinery.^4^ Positive-sense RNA (ssRNA+) viruses, such as dengue and SARS-CoV-2, use their genomes directly as mRNAs to recruit ribosomes and translate viral proteins, while negative-sense RNA (ssRNA-) viruses rely on their RNA-dependent RNA polymerase (RdRp) to synthesize complementary positive-sense RNAs for translation.^5^^,^^6^^,^^7^ Thus, to establish a productive infection, the virus hijacks the host’s cellular machinery for viral protein expression, genome replication, and virion assembly.

Being an obligate intracellular parasite, a viral genome faces significant challenges when competing with the host’s endogenous transcripts. As a result, viruses have developed counter strategies to overcome these defences, ensuring that the translation of viral proteins remains uninterrupted.^8^^,^^9^ Many ssRNA+ viruses enhance their replication efficiency by inducing host shutoff, a process that selectively suppresses host protein synthesis. This mechanism shifts the translational machinery’s focus entirely toward viral mRNAs, ensuring efficient production of viral proteins.^10^ For instance, SARS-CoV-2 achieves host shutoff by degrading host mRNAs via its non-structural protein NSP1, which blocks mRNA entry into ribosomes.^11^^,^^12^ Similarly, dengue virus co-opts the host’s ribosomes by directing them to the endoplasmic reticulum, where viral mRNA translation is compartmentalized, effectively side-lining host mRNA translation.^3^^,^^13^ These strategies exemplify how ssRNA+ viruses prioritize their replication while undermining host defences.

Dengue virus (DENV) rely on efficient transcription and translation mechanisms to maximize the output of their compact genomes. These processes are tightly linked, with translation often shaped by the structure and sequence of the viral RNA.^14^^,^^15^ A comprehensive understanding of these mechanisms requires the integration of transcriptomic and translatomic approaches. RNA sequencing (RNA-seq) provides an unbiased global overview of the transcriptome, identifying the abundance and distribution of RNA transcripts, while ribosome profiling (Ribo-seq) reveals active translation by mapping ribosome occupancy on the mRNA. Together, these genome-wide techniques offer critical insights into viral gene expression, particularly translational efficiency (TE)—the ratio of ribosomal footprints to the mRNA abundance.^16^^,^^17^

The interplay between RNA-seq and Ribo-seq data also sheds light on the potential codon bias, a key factor in translational regulation. Codon bias, the preferential use of specific codons, allows RNA viruses to optimize translation by adapting their codon usage to the host’s tRNA pool.^18^^,^^19^^,^^20^ By exploiting codons corresponding to abundant host tRNAs, viruses like DENV enhance their translational efficiency, enabling rapid protein synthesis critical for their replication.^21^ This adaptation is reflected in the ribosome profiling, which reveals ribosomal occupancy at the specific codons and highlights regions of high translational activity.^22^

For ssRNA+ viruses with constrained genome sizes, codon optimization is only one of the several strategies employed to maximize translation. Recent studies, including those on SARS-CoV-2, have revealed the existence of non-canonical translational mechanisms, such as the use of internal or upstream open reading frames (iORFs/uORFs), ribosomal stalling, and alternative frame usage.^23^ These mechanisms allow viruses to expand their coding potential and regulate protein synthesis in response to the host conditions. Exploring such mechanisms in DENV could uncover how the virus tailors its genome to efficiently use host translational machinery while evading antiviral responses. For DENV, these mechanisms are particularly relevant due to its small genome (∼11 kb), which must encode a wide array of proteins essential for replication, immune evasion, and virion assembly as it shuttles between two hosts, *Aedes* mosquito and human. Alternate ORFs can produce accessory proteins that assist in immune evasion by modulating host responses or hiding viral antigens from detection, enhancing the virus’s survival. The dengue genome is known to consist of a single ORF that translates into a polyprotein precursor that is cleaved into functional proteins. Thus, to investigate non-canonical translational mechanisms in the dengue genome, we utilized RNA-seq data obtained from the hospital-admitted dengue-positive patient samples. This approach allowed us to examine unbiased viral genome coverage patterns and identify regions of interest for potential non-canonical translation. By comparing clinical RNA-seq data with independent paired Ribo-seq and RNA-seq datasets for DENV-2 serotype, we explored the potential translational strategies employed by the DENV to optimize its genome usage. Special attention is given to peaks in coverage that may correspond to putative ORFs or other features indicative of alternative translation mechanisms. Insights gained from this analysis could reveal novel aspects of DENV biology, such as its ability to employ regulatory or adaptive translational strategies, offering new avenues for therapeutic intervention. Although not investigated in this study, it would be worth checking in future studies whether these DENV genome innovations differ between its stay within the *Aedes* and human host.

## Results

### Base composition across dengue genome

In this study, we recruited 112 dengue-positive patients admitted to Max Super Speciality Hospital, New Delhi, India, who were confirmed NS1 antigen-positive by RT-PCR. Blood samples were collected from these patients, and RNA-seq libraries were prepared to investigate host-pathogen interactions. In a previous study, we examined the effects of viral infections on the host translational machinery and its impact on the alternative splicing patterns and protein-coding capacity of the host.^24^ In the current study, we aimed to understand the potential evolutionary strategies employed by the DENV genome to ensure its survival and effective translation within the host cells. Additionally, we investigated how the virus may maximize the utility of its limited genome size, leveraging mechanisms such as codon optimization and creation of novel genes, principally by +1 frameshifts giving rise to small internal ORFs (iORFs), to enhance replication and protein production.^25^

**Figure 1:**
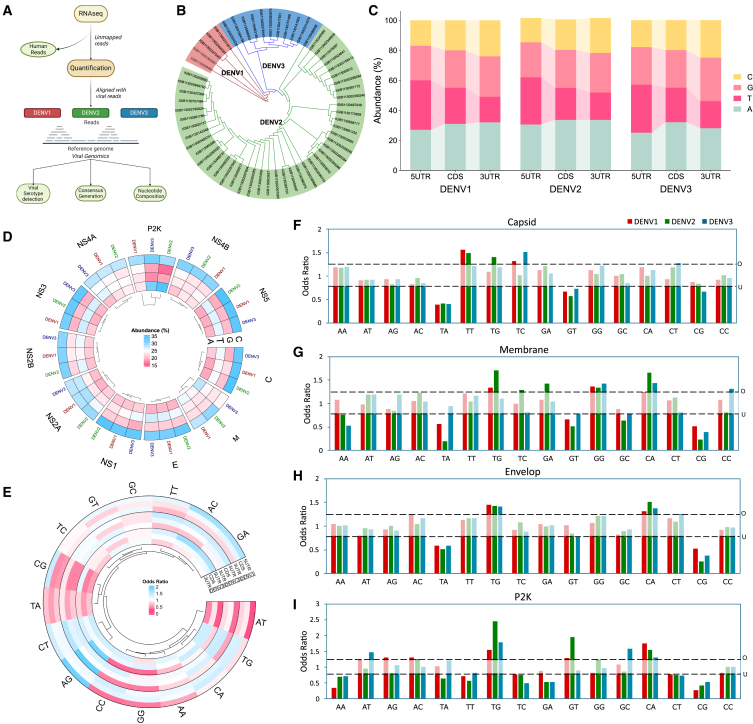
Nucleotide and dinucleotide composition analysis of DENV serotypes (A) Schematic representation of the viral component extraction from the RNA-seq data of the hospital admitted clinical samples. (B) Phylogenetic tree of the DENV serotypes derived from the clinical samples, with DENV-1 in red, DENV-2 in green, and DENV-3 in blue. (C) Comparison of the nucleotide composition across the 5′ UTR, CDS, and 3′ UTR regions in the DENV-1, DENV-2, and DENV-3 serotypes. (D) Gene-wise nucleotide composition across the DENV-1, DENV-2, and DENV-3 serotypes. (E–I) (E) Dinucleotide composition in the 5′ UTR, CDS, and 3′ UTR regions across the DENV-1, DENV-2, and DENV-3 serotypes, represented as odds ratios. Dinucleotide composition across the DENV-1, DENV-2, and DENV-3 serotypes in (F) Capsid, (G) Membrane, (H) Envelope, and (I) p2k genes, represented as odds ratios. See also Figures S1–S3. Data are represented as odds ratios.

RNA-seq reads that were unmapped to the human host genome were quantified against the DENV genomes and consensus FASTA sequences were generated (Table S1). These were then analyzed to determine the nucleotide and amino acid composition of the viral genome (Figure 1A). From the consensus FASTA sequences generated, we performed phylogenetic analysis to identify the serotype of the sequences captured from the clinical samples. Of the 112 samples, we captured high viral reads (>50X depth and >99% coverage) in 58 samples. Among these 58 samples, we observed DENV-2 to be the most prominent serotype (*n* = 42), followed by DENV-3 (*n* = 11) and DENV-1 (*n* = 5) (Figure 1B). It is also important to note that in India DENV-2 is the dominant serotype causing infections every year.^26^

The initial step of replication of a +ssRNA virus like dengue is translation. Therefore, to understand translational dynamics in dengue virus, we first explored the genetic variability within the viral genome that influences protein expression, genome stability, and the formation of secondary structures. Using the consensus sequences, the base composition of the dengue genome was calculated across three key regions: 5′ and 3′ untranslated regions (UTR), and the coding sequence (CDS) for the dengue serotypes. The analysis revealed a pervasive distribution of AT/GC among DENV-1, DENV-2, and DENV-3 (Figure 1C). Notably, significant differences in AT/GC compositions were observed across genomic positions from the 5′ UTR to 3′ UTR in DENV-1 (p val = 0.008) and DENV-3 (p val = 0.006).

Adenine is the most abundant base in the DENV genome, with its frequency increasing from 5′UTR to CDS and remaining consistent in the 3′UTR. In contrast, thymine shows a significant decline from the 5′ to 3′ UTR (*p* value = 0.02). Guanine and cytosine both show a gradual increase, with cytosine showing a statistically significant rise (p val = 0.02). In the 5′ UTR, nearly 60% of the bases are adenine (A) whose content is highest in DENV-2 (30.2%), compared to 27% in DENV-1 and 25% in DENV-3. Conversely, thymine (T) is most abundant in DENV-1 (33%), followed by DENV-3 (32%) and DENV-2 (31.3%). Guanine (G) and cytosine (C) content are relatively stable, although DENV-3 shows slightly higher G content at 25%, compared to 23% in DENV-1 and 22.9% in DENV-2. It is important to note that these small genome percentage changes are important when we visualize in the context of the number of base pairs vis-à-vis the DENV genome size of ∼11kb. Elevated GC content indicates a higher potential for the stable secondary structures in the 3′ UTR, which could enhance the stability of the viral genome and its interaction with the host or viral proteins. Such stability is crucial for genome circularization, a mechanism essential for flavivirus replication.^27^^,^^28^

At the codon level, A emerged as the dominant nucleotide across all positions (N1, N2, and N3), with a significantly higher representation at N3 position in all the serotypes (p val = 0.02) (Figure S1A). Likewise, G was also significantly enriched at N3 (p val = 0.02) In contrast, T and C were significantly less prominent, particularly at N3 (p val = 0.02). Gene-wise analysis revealed that the frequency of T and C was significantly lower compared to A (p val <0.001), while no difference in the nucleotide composition was observed between DENV genes (Figure 1D). Viruses are observed to adapt the codons bias of highly expressed host genes in the specific tissue they infect^29^ with the likely potential of optimizing translational efficiency by aligning with the host’s tRNA pool to enhance the speed and accuracy of the protein synthesis.^30^ The increased prevalence of A and G bases at third position may play a role in this process.

### Relative dinucleotide composition

Next, we explored the composition of dinucleotides motifs across the genomic positions. The dinucleotide composition of dengue virus serotypes reveals region-specific biases/patterns. In the 5′ UTR - AG, AC, GA, and CT were consistently overrepresented (odds ratio >1.25) while AT, CC, and GG were the underrepresented dinucleotides (odds ratio <0.78) in all the three serotypes, with TT additionally overrepresented in the DENV-2 and DENV-3 serotypes and CG in the DENV-1 (Figure 1E) overrepresented. In the CDS region, TG and CA emerged as the only motifs universally overrepresented across all the serotypes, while TA and CG were notably underrepresented. The 3′ UTR represented a gradually different perspective across the serotypes. TG and CC were overrepresented in all the serotypes while CG, TA, and TT were underrepresented, with CT showing higher presence in the DENV-2 and DENV-3. Additionally, AG was uniquely overrepresented while AT was under-represented in the DENV-3, highlighting serotype-specific differences in the dinucleotide preferences. These dinucleotide biases reflect a delicate balance between optimizing viral functionality and evading host defences.

We further analyzed the dinucleotide composition across specific codon positions—(NN)_12_, (NN)_23_, and (NN)_31_—within the CDS region and the structural and non-structural genes and to uncover potential patterns of codon usage and selective pressures shaping the dengue genome. Among the 16 dinucleotides, TG was consistently overrepresented, whereas GT, TA, and CG were underrepresented at the (NN)_12_ and (NN)_23_ positions across all the three serotypes, indicating a conserved trend in their distribution (Figure S1B).^31^ Conversely, AT was notably underrepresented at the (NN)_31_ position in all the serotypes, however at the gene-level it shows optimal usage patterns across both structural and non-structural genes (Figures 1F–1I). This may indicate selective pressure to avoid specific motifs that could affect RNA secondary structure or translation dynamics. Despite A being the most abundant nucleotide in the genome, only the CA motif is found to be over-represented in the DENV genes (Figure S2). Overall, we observe a similar dinucleotide distribution across genes except for p2K, possibly driven by functional constraints to maintain overall genomic stability.^32^

### Codon usage biasness

The dengue codon usage pattern is shaped by its nucleotide composition, as revealed by relative synonymous codon usage (RSCU) analysis, as an adaptation to the human host. Among the 59 codons, only six codons were significantly overrepresented with RSCU values > 1.6, five of which ended in A. Conversely, nine codons were strongly underrepresented with RSCU values < 0.6, five of which ended in G. Overall codon distribution in dengue virus reveals a strong preference of A-ending codons and a strong codon usage bias toward the G/C -ending codons (Figure S3A). Also, among the serotypes, DENV-2 shows the most adaptation to the host’s translational machinery, as indicated by its effective number of codons (ENc = 49.27), compared to the DENV-1 (ENc = 50.5) and DENV-3 (ENc = 50.04), suggesting a slightly stronger codon usage bias in DENV-2 (Figure S3B).

### Assessing viral adaptability

To further assess viral adaptability, the Codon Adaptation Index (CAI) was calculated for the dengue virus polyprotein, revealing moderate adaptation for all the serotypes (DENV-1: 0.66, DENV-2: 0.68, DENV-3: 0.67) (Figure S3C). The Relative Codon Deoptimization Index (RCDI) showed that DENV-2 had the lowest value (1.43) indicating marginally better adaptation (Figure S3D). The SiD values were similar across all the serotypes, suggesting a comparable effect of the human host on the virus (Figure S3E).

However, the correspondence analysis using RSCU value underscores the complex interplay between the human host-driven selective pressures and intrinsic constraints of the dengue virus. The distinct clustering of DENV-3 in the first quadrant suggests unique evolutionary pressures. In contrast, the shared clustering of the DENV-1 and DENV-2 in the fourth quadrant indicates overlapping codon usage patterns (Figure S3F).

Overall, the correspondence analysis suggests that the virus may selectively optimize certain regions to improve replication and expression within the human host. The first axis accounted for 90.4% of the variance, while the second axis explained 5.7%, indicating that the primary axis governs codon usage in dengue virus. The distribution of synonymous codons ending in A and some G-ending codons emerged as the potential key contributing factors for variance along both axes.

### Differential genome coverage

**Figure 2:**
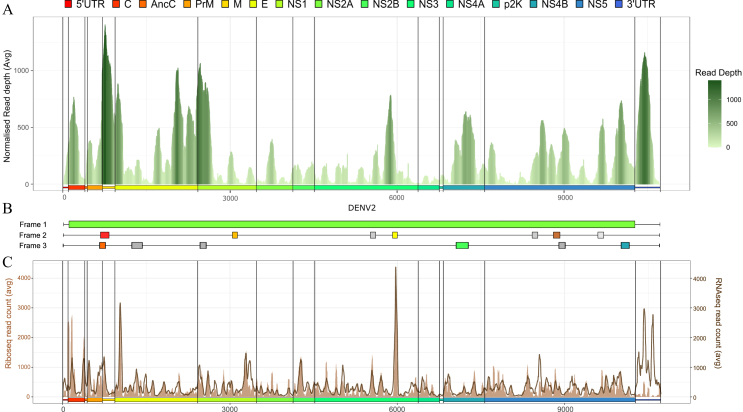
Genome coverage and translation dynamics of the DENV-2 genome (A) The area plot illustrates the normalised read depth across the DENV-2 genome. (B) A graphical representation of the potential open reading frames (ORFs) predicted across the viral genome in frames 1, 2, and 3. (C) A dual-axis line plot showing read density from the Ribo-seq and RNA-seq datasets, with the primary axis representing Ribo-seq coverage and the secondary axis representing paired RNA-seq coverage across the DENV-2 genome. See also Tables S1 and S2A–S2C, Figures S4 and S7. Data are represented as mean normalised abundance.

**Figure 3:**
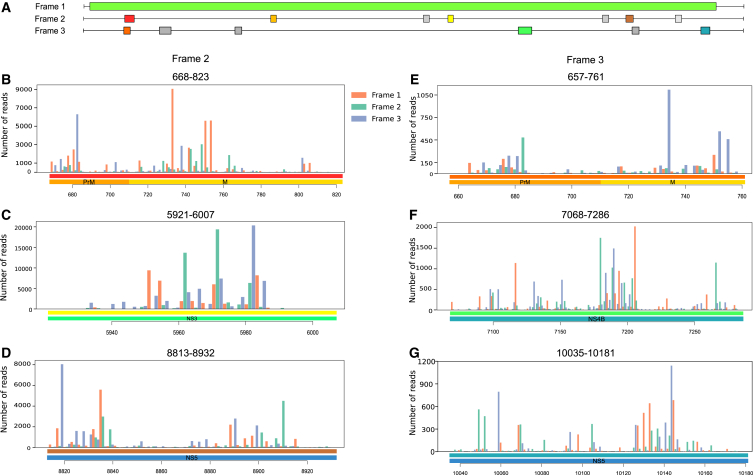
Translational activity across predicted iORFs (A) Graphical representation of the DENV-2 reading frames and predicted iORFs in frame 2 and frame 3 in color. (B–D) Bar plots display ribosomal read profiles predicted for potential iORFs in frame 2 using the Ribotricer tool, with bars representing the position of ribosomal reads in frame 1 (red), 2 (green), and 3 (blue) across the ORFs. (E–G) Similar bar plots represent potential ORFs in frame 3, with the gene track below indicating the position of predicted ORF in the DENV-2 genome. See also Tables S2D and S2E, Figure S5. Data are represented as normalized reads counts.

Thus far, we have examined the viral codon usage patterns and adaptation metrics to gain insights into how dengue virus serotypes may adjust their genomes for efficient replication within the differential human host microenvironment. Building upon this, we analyzed the coverage dynamics across all the DENV samples to further explore the impact of these adaptive strategies on the viral translation and replication. Interestingly, the DENV-2 genome showed a unique profile compared to the DENV-1 and DENV-3.The DENV-2 profile revealed pronounced peaks and troughs in the RNA-seq read coverage across the dengue genome, while DENV-1 and DENV-3 had relatively consistent coverage across the genome (Figure 2A; Figure S4). This variability in coverage is indicative of differences in expression levels among various viral genes. Distinct peaks were observed around the M protein, at the start and end of the E protein, at the beginning of NS1, and in the scattered regions of the NS3, NS4, and NS5 genes in the DENV-2 serotype (Figure 3A). A particularly high read density was noted around the 3′ UTR. The peaks observed in the genome coverage indicate regions of significant translational activity, prompting an exploration/elucidation of the potential ORFs. Considering the relatively small sample size in this study for the DENV-1 and DENV-3 serotypes and uniform coverage patterns, these were not included in the further analysis. The consensus FASTA sequence of the DENV-2 genome derived from the clinical samples was uploaded to the NCBI ORFfinder tool, where putative ORFs in all the three positive reading frames were analyzed. A total of 26 ORFs were predicted across the three frames, with Frame 1 containing a single ORF as the primary reading frame. After filtering based on a read density greater than 100, 14 ORFs were identified: one in Frame 1, seven in Frame 2, and six in Frame 3 (Figure 2B) (Table S3A).

### Non-canonical translation events

To investigate the translation across the predicted small internal ORFs (iORFs) in the dengue genome, we analyzed an independent publicly available dengue virus Ribo-seq dataset with matching/paired RNA-seq data (GSE69602) from the Huh-7 (human hepatocarcinoma cells; ATCC) infected cell-line corresponding to the DENV-2 serotype from a previously published study by Reid et al.^33^ Samples from the ER fraction of the study were analyzed for a comparison of the dengue virus genome coverage captured from the paired RNA-seq and Ribo-seq Huh-7 datasets (Table S3C). The comparison revealed that ∼70% of the peaks overlapped between the paired RNA-seq and Ribo-seq datasets from the cell-line, indicating regions of active translation. However, notable differences were observed near the 3′ UTR in the RNA-seq coverage profile and ribosome profile (Figure 2C). Additionally, a comparison of the viral reads captured from the clinical RNA-seq data (from in-house data) with the RNA-seq (from Huh-7 cell-line data) indicated that ∼20% of the coverage patterns were similar. Further analysis focused on evaluating translation activity from the Ribo-seq dataset.

As we did with the (in-house clinical) RNA-seq dataset, we predicted iORFs using the ORFfinder tool for the DENV-2 consensus sequence extracted from the (Huh-7 cell-line) RNA-seq dataset. Upon predicting iORFs, the data analysis revealed 21 putative iORFs of which 13 overlapped between the RNA-seq (clinical samples) and RNA-seq (Huh-7 cell-line) datasets (Table S3D). We next employed the Ribotricer tool to detect and predict translation around these predicted putative internal ORFs (iORFs). Of the 13 common iORFs, only 9 demonstrated translational evidence in the Ribo-seq dataset. These iORFs were distributed across Frame 2 (6 ORFs) and Frame 3 (3 ORFs), highlighting potential differential usage of translational frames within the DENV-2 genome (Figure 3A) (Table S3E).

In Figures 3B–3D, the ribosome profiles corresponding to Frame 2 iORFs are shown, where regions like 5921–6007 within the NS3, and 668–823 in the M protein protein exhibit a clear alignment between the RNA-seq peaks and Ribo-seq read distribution. This suggests active translation around these loci. Specifically, the read distribution in Frame 2 aligns with the peaks observed in the RNA-seq dataset (Figures S6A–S6C). Similarly, Figures 3E–3G illustrates the ribosome profiles for Frame 3 iORFs. M protein also shows peak in Frame 3 around 657–761. The consistent presence of Ribo-seq reads around peaks in the RNA-seq reads suggests these regions are also potentially translated, albeit with reduced efficiency or governed by the specific regulatory mechanisms. Overall, the mixed ribosome signals across these ORFs may reflect varying levels of translation efficiency or selective ribosome engagement, pointing to potential regulatory roles of these iORFs in the DENV-2 biology within the human host.

**Figure 4:**
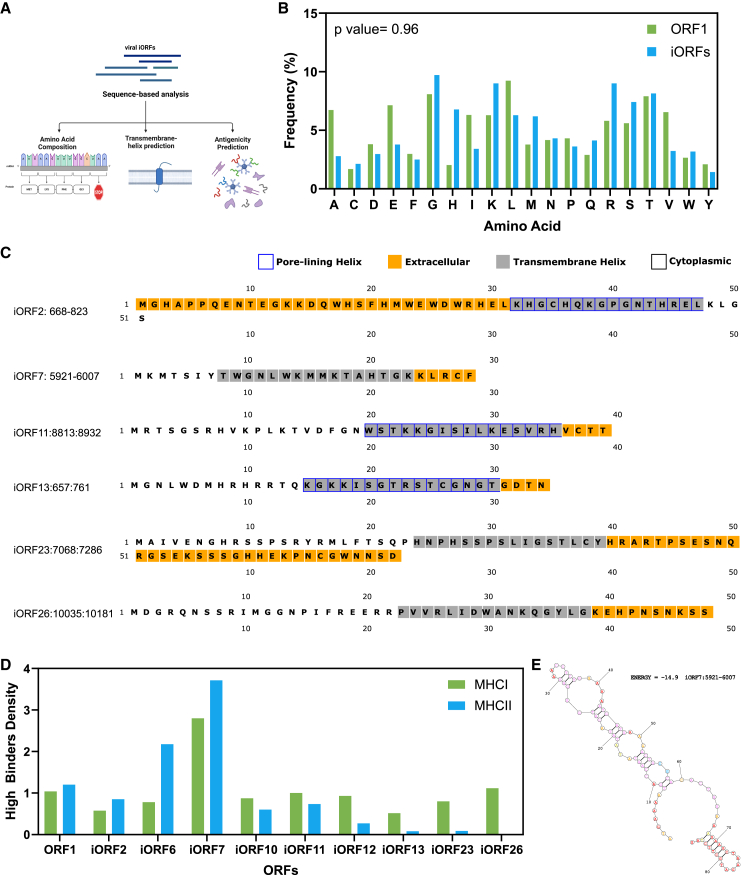
Sequence-based analysis of iORFs for functional interpretation (A) Graphical representation of methods/analysis performed on iORFs. (B) Comparison of amino acid composition between the long ORF1 and short iORFs. (C) Transmembrane helix prediction across the iORFs, the sequences colored in yellow represent extracellular regions, regions in gray represent transmembrane helix, regions in white are cytoplasmic, while regions with blue border are predicted to form pore-lining helix. (D) High binding peptide density for the MHC-I and MHC-II molecules in each ORF. (E) Secondary structure of iORF7. See also Table S3, Figure S6. Data is represented as proportions.

We further analyzed the selected iORFs in both the frames using sequence-based methods to characterize their general properties and gain insights into their potential functions (Figure 4A).^34^ We first compare the amino acid composition of these iORFs against the canonical long ORF1 using the Uniprot ProtParam tool. We do not find any significant differences between the amino acid composition of both the long ORF1 and short iORFs (Figure 4B). This indicates functional parity between the long and short ORFs, suggesting that similar selective pressures govern amino acid composition in the iORFs, and could play biologically relevant roles. Using MEMSAT-SVM, we analyzed the transmembrane helix content of predicted iORFs in the viral genome. While none of these sequences show signal peptides, the iORFs were predicted to contain a transmembrane helix spanning residues (Figures 4C and S6C–S6E). Further analysis revealed a moderate likelihood of some of the iORF helices (iORF2, iORF6, iORF10, iORF11, iORF12, and iORF13) being pore-lining. These findings indicate that some iORF may encode a membrane-associated protein with potential roles in the viral entry, intracellular transport, or host-virus interactions. We evaluated the antigenicity and potential immune recognition of these peptides using the NetMHC and NetMHC-II web tools. These platforms predict MHC class I and class II binding affinities, respectively, enabling the identification of peptides with a high likelihood of being recognized by the host immune system (Table S4). The density of high-binding peptides for each of the iORFs was analyzed, revealing that iORF7 exhibited the highest density of both MHC-I and MHC-II binding sites (Figure 4D). This suggests that iORF7 peptide may have a role in immune recognition and interaction with the host. Examining the secondary structure of iORF7, we identified a stable paired region accompanied by the accessible loops (Figure 4E). These loops present an ideal target for immune recognition, likely contributing to the generation of peptides with high binding affinity to MHC molecules.

## Discussion

In this study, we explored and tried to elucidate the potential evolutionary strategies employed by the dengue virus to optimize its genome for replication and translation within the human host. By analyzing blood samples from the 112 hospital-admitted DENV-positive patients, we investigated the serotype-specific nucleotide composition and its potential impact on the translation efficiency, genome stability, and human host-virus interactions. Our findings highlight differences in the base composition that reflect adaptations to balance structural stability with the functional requirements of translation and replication.

The region-specific differences in the base composition highlight their distinct roles in viral genome replication and translation.^35^ For example, the increased G/C content in the 3′ UTR may improve RNA stability under human host-induced stress, with this region encoding viral non-coding RNA (ncRNA) that interacts with the TRIM25 protein, a critical regulator of the type I interferon response. By stabilizing this interaction, dengue virus may suppress the host’s antiviral immune response, thus promoting viral replication and survival.^36^ The A-rich 5′ UTR may facilitate weaker secondary structures, allowing for the efficient initiation of protein synthesis by enhancing ribosome binding and scanning.^37^ The 5′ UTR of DENV can act as an internal ribosome entry site (IRES), enabling initiation toward ORF1 during early infection which expresses the viral polyprotein.^38^ In ssRNA(+) viruses, adenine (A) is typically the most abundant nucleotide, driven by both mutational bias and selective pressures. However, flaviviruses deviate from this pattern, exhibiting a unique nucleotide composition enriched in both adenine (A) and guanine (G), which contrasts with the trends seen in other ssRNA(+) viral families due to the alternative vector-borne aspects of their viral life cycle and replication mechanisms.^39^

The dinucleotide composition exhibited significant variation across the different genomic regions, yet it remained largely conserved among the dengue virus serotypes. The relative abundance of dinucleotides can significantly impact the codon usage pattern in the virus genome.^40^ Notably, the overrepresentation of TpG and CpA motifs, along with the underrepresentation of CpG and TpA, was also observed in other ssRNA(+) viruses such as influenza A virus (IAV) and SARS-CoV-2.^41^^,^^42^ The suppression of CpG motifs in many RNA viruses is a well-documented strategy to evade host defense mechanisms, especially the methylation processes that target cytosine residues.^43^^,^^44^ This results in deamination and mutations to thymine, reducing immune recognition.^45^^,^^46^ Recent studies have shown that CG dinucleotide suppression can further enhance this evasion by preventing recognition of viral RNA as non-self by the host immune system, allowing the virus to better avoid immune detection.^47^ This mechanism has important implications for viral adaptation and immune response strategies. Additionally, TpA motifs may be underrepresented due to their susceptibility to degradation by host RNases, which can destabilize RNA molecules containing this dinucleotide.^48^ This pattern demonstrates evolutionary adaptations to selection pressures from the human host genome, with the virus mimicking the host’s dinucleotide patterns to improve translational efficiency and evade the antiviral response, thereby supporting viral persistence and success within the host micro-environment.^46^

The pattern of dinucleotide usage in the DENV-2 suggests a broader strategy where the virus optimizes its codon usage to enhance translational efficiency while evading the host immune system. This optimization is shaped by both the mutational and purifying selection pressures, as highlighted in a study by Ramirez et al.^49^ Dengue virus leverages the host’s ER-ribosomes for translation, with codon usage carefully balancing the precise matching required for efficient translation and fitting the diverse tRNA pools across the different hosts.^33^

To assess the extent of adaptation, we computed ENc values, which revealed that among the serotypes, DENV-2 is most adapted to the host (human), resulting in greater expression efficiency compared to the other serotypes. This potential adaptation aligns with the increased severity and prevalence of the DENV-2 infections, as observed in our data, where DENV-2 was the predominant serotype. The suboptimal codon-usage compared to the host codons for viral gene expression, could represent a host-pathogen co-evolutionary strategy designed to delay the activation of the host immune response.^50^ These observations imply that specific genomic regions may have been shaped by the evolutionary pressures to enhance translation efficiency.

Next, we analyzed the coverage profile of viral genome from the RNA-seq dataset for 43 DENV-2 hospital-admitted patients. The observed peaks and troughs in the read coverage across the dengue virus genome highlight differential expression levels among the viral genes, reflecting transcriptional and translational priorities. The high read density in the 3′ UTR region emphasizes the role of subgenomic flavivirus RNAs (sfRNAs) in regulating viral translation and replication, consistent with its known functions in genome stability and interaction with the host immune evasion.^51^ Pronounced peaks near the M protein, E protein termini, NS1, and regions of NS3, NS4, and NS5, suggest a targeted expression strategy, potentially correlating with viral survival, assembly, or immune evasion. Upon exploring the codon usage patterns in these regions, no significant differences were observed between the peaks and the troughs (Figure S5). Interestingly, several of these peaks corresponded to putative internal ORFs (iORFs) within the DENV virus genome, highlighting potential non-canonical translation mechanisms in play. While dengue virus genome is known to translate using a single polyprotein, which is later cleaved to form structural and non-structural genes, the overlap between several peaks and predicted iORFs within Frames 2 and 3 suggests a possible weak translation in these two reading frames.

**Figure 5:**
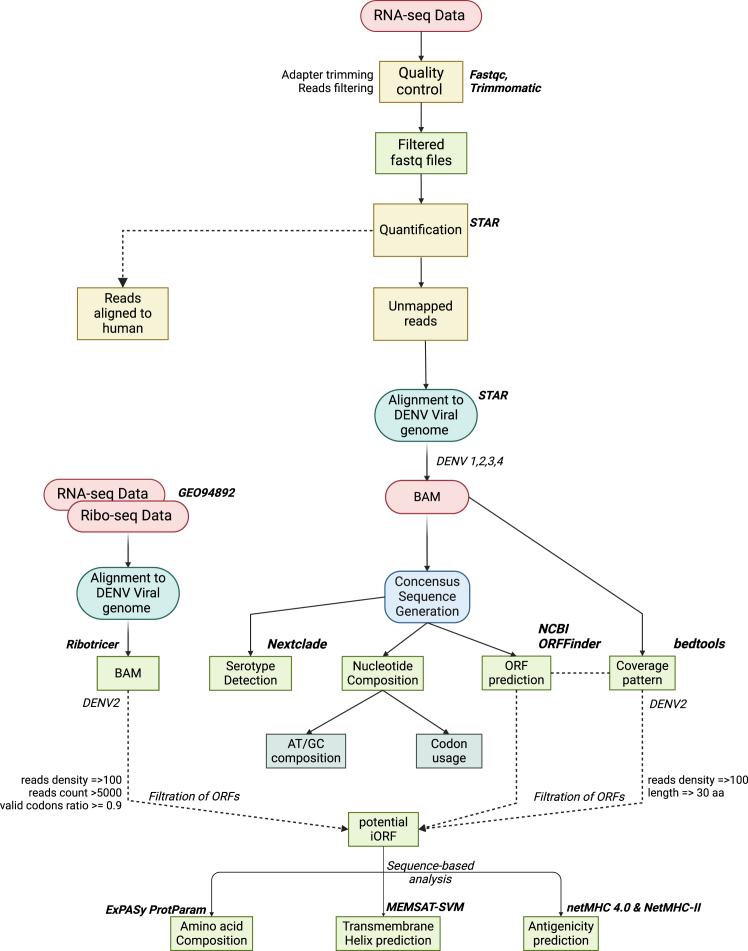
The flowchart represents the computational pipeline used in the study to understand viral genome from the RNA-seq data of dengue patient samples

To assess whether the genome coverage pattern was consistent across the different and independent datasets, we compared RNA-seq data from the cell line study with the clinical RNA-seq data (Figure 5). This comparison revealed host-specific differences in the potential dengue virus translation, with about 20% overlap in the coverage patterns across both datasets, suggesting that translation efficiency or the utilization of internal ORFs may vary depending on the cellular environment. The significant overlap (∼70%) between the Ribo-seq and RNA-seq data indicates active translation in these regions, highlighting their functional importance in the dengue virus genome.

The identification of 13 putative internal ORFs (iORFs), with 9 showing translational activity, supports the idea of weak yet probable non-canonical translation events. This suggests that DENV-2 may utilize iORFs for regulatory or functional purposes, potentially involved in the immune evasion, protein synthesis regulation, or other survival strategies. The alignment of ribosome profiles with RNA-seq peaks, particularly in regions like 5921–6007 (iORF7) within NS3, suggests these loci are plausibly actively translated. Although the translation signal in Frame 3 is weaker, it still suggests some degree of potential activity, which may reflect selective ribosome engagement or lower translation efficiency. The predicted iORFs, particularly those in the NS3 gene (e.g., ORF6 and ORF7), exhibit high binding affinities for MHC molecules.^34^^,^^52^ Peptides with strong binding affinities to MHC molecules are more likely to be recognized by cytotoxic T cells (CD8^+^ T cells), making these small ORFs potentially critical for the host immune system’s ability to detect and respond to DENV infection.^53^ This suggests that small DENV products could have important immunological implications-such as enabling recognition by the immune system,^54^ modulate host immune response by interacting with immune signaling pathways,^55^ or encode immunodominant peptides shaping viral evolution by selective mutational pressures.^56^ Such findings would emphasize the dual importance of sORFs as both immunological targets and modulators, with significant implications for understanding viral evolution, pathogenesis, and the design of vaccines and therapies.

Other ssRNA viruses such as SARS-CoV-2 are also shown to exploit upstream ORFs for enhancing its coding efficiency.^23^ Such potential ORFs are not explored in the dengue virus, possibly due to the weaker/mixed signals from such translation events. However, this study shows that there is perhaps some evidence of pervasive/non-canonical translational events in the dengue virus genomes. Future research is required to determine whether these iORFs are a result of leaky translation in the human host genome or underscore stress-related translation mechanisms, potentially contributing to the viral persistence and pathogenesis.

In conclusion, this study highlights the potential intricate evolutionary strategies employed by the dengue virus to adapt its genome for efficient replication and translation within the human host. The codon and dinucleotide usage patterns across the dengue virus serotypes reveal a fine balance between optimizing translational efficiency and evading host immune responses. Serotype-specific adaptations, particularly in DENV-2, underscore its enhanced replication efficiency and association with increased disease severity. The analysis of dengue virus genome coverage and the identification of putative internal ORFs suggest that non-canonical translation events may play a role in viral survival, immune evasion, and protein synthesis regulation. These findings advance our understanding of dengue virus evolution and human host–virus interactions, providing a foundation for future investigations into novel therapeutic targets and the functional implications of non-canonical translation mechanisms in viral pathogenesis.

### Limitations of the study

Ribo-seq studies on clinical samples still faces several challenges. Therefore, integrating paired Ribo-seq and RNA-seq data from clinical samples would enhance iORFs validation. Further research is required to explore the role of iORFs in host immune response and viral replication, thereby modulating dengue disease severity and outcomes.

## Resource availability

### Lead contact

Further information and requests for resources and reagents should be directed to and will be fulfilled by the lead contact, Rajesh Pandey (rajesh.p@igib.res.in).

### Materials availability

This study did not generate new unique reagents and material.

### Data and code availability

• RNA-seq datasets presented in this study has been deposited at the NCBI-SRA and are publically available as of the date of publication. Accession numbers are listed in the key resources table. All the data reported in this paper will be shared by the lead contact upon request. under the BioProject PRJNA1071729.
• This paper does not report the original code.
• Sample-wise accession numbers are additionally provided in Table S1.
• Any additional information required to re-analyze the data reported in this paper is available from the lead contact upon request.

## Acknowledgments

The authors duly acknowledge all the dengue patients who participated in the study. The authors further recognize Aanchal Yadav and Aparana Swaminathan for their contributions to sample isolation, library preparation, and sequencing data collection in this study. Authors acknowledge the help and support from Dr Bharti Kumari toward facilitation as research manager and coordination with the funders. Authors acknowledge the support of Anil Kumar and Nisha Rawat toward dengue sample transport and sample management. P.D. acknowledges the CSIR for his Research Fellowship. This research was funded by 10.13039/100000865Bill and Melinda Gates Foundation, (grant no. - INV-033578) and 10.13039/100000877Rockefeller Foundation, (grant no. - 2021 HTH 018) to RP.

## Author contributions

P.M.: Formal analysis, visualization, investigation, and writing – original draft. P.D.: Investigation, experiments, literature review, writing – original draft. R.P.: Conceptualization, writing – review and editing, supervision, and funding acquisition. All authors discussed the results and contributed to the final manuscript.

## Declaration of interests

The authors have declared that no conflict of interest exists, and the funding body did not have any role in study design, experiments conducted, or interpretation of the results.

## Declaration of generative AI and AI-assisted technologies in the writing process

During the preparation of this work, the author(s) used Grammarly to improve the readability of the manuscript. After using this tool, the author(s) reviewed and edited the content as needed and take(s) full responsibility for the content of the publication.

## STAR★Methods

### Key resources table

**Table:** 

REAGENT or RESOURCE	SOURCE	IDENTIFIER
**Chemicals, peptides, and recombinant proteins**		

Blood RNA extraction	QIAmp RNA Blood mini kit, Qiagen	Cat. No. 52304
TruSeq Stranded Total RNA Library Prep Globin	Illumina	Cat. No. 20020612
AMPure XP	Beckman Coulter	Cat. No. A63881
Agencourt RNAClean XP Kit	Beckman Coulter	Cat. No. A63987
Qubit dsDNA HS Assay kit	Thermo Fisher Scientific	Cat. No. Q32854
Agilent 2100 Bioanalyzer	Agilent	Cat. No. 5067-4626
NanoDrop 2000 Spectrophotometer	Thermo Fisher Scientific	Cat. No. ND2000CLAPTOP
NextSeq 2000 P2 reagent kit (300 cycles)	Illumina	Cat. No. 20046813

**Deposited data**		

RNA-seq data	This study, NCBI Sequence Read Archive (SRA) database. Sample-wise accession numbers are additionally provided in Table S1.	SRA: PRJNA1071729
Ribo-seq paired RNA-seq data	Generated by Reid et. al^33^ submitted to the Gene Expression Omnibus (GEO)	GEO:GSE69602

**Software and algorithms**		

FastQC	Andrews et al.^57^	https://www.bioinformatics.babraham.ac.uk/projects/fastqc/
Trimmomatic v0.39	Bolger et al.^58^	https://github.com/timflutre/trimmomatic
Prism 9	GraphPad	https://www.graphpad.com/
Trim Galore	Krueger^59^	https://github.com/FelixKrueger/TrimGalore
STAR 2.7.10b	Dobin et al.^60^	https://github.com/alexdobin/STAR
Samtools 1.15.1	Danecek et al.^61^	https://github.com/samtools
Bcftools 1.10.2	Danecek et al.^61^	https://github.com/samtools/bcftools
Seqtk 1.4-r122	NA	https://github.com/lh3/seqtk
Nextclade	Nextstain	https://clades.nextstrain.org/
NGphylogeny.fr	Lemoine et al.^62^	https://ngphylogeny.fr/
MAFFT version 7	Katoh et al.^63^	https://mafft.cbrc.jp/alignment/server/index.html
FastME	Lefort et al.^64^	https://gite.lirmm.fr/atgc/FastME/
iTOL v7 (Interactive Tree Of Life)	Letunic et al.^65^	https://itol.embl.de/
Codon Usage Database	Nakamura et al.^66^	https://www.kazusa.or.jp/codon/
vhcub v1.0.0	Anwar et al.^67^	https://github.com/AliYoussef96/vhcub
FactoMineR v2.11	Husson et al.^68^	https://github.com/husson/FactoMineR
factoextra v1.0.6	Lê et al.^69^	https://github.com/kassambara/factoextra
ORFfinder	NCBI	https://www.ncbi.nlm.nih.gov/orffinder/
sratoolkit	NCBI	https://github.com/ncbi/sra-tools
Ribotricer v1.4.0	Choudhary et al.^70^	https://github.com/smithlabcode/ribotricer
Bedtools v2.29.1	Quinlan et al.^71^	https://github.com/arq5x/bedtools2
ProtParam	ExPASy	https://web.expasy.org/protparam/
MEMSAT SVM	UCL Bioinformatics Group	https://github.com/psipred/MemSatSVM
netMHC 4.0	DTU Health Tech	https://services.healthtech.dtu.dk/services/NetMHC-4.0/
netMHC-II 2.3	DTU Health Tech	https://services.healthtech.dtu.dk/services/NetMHCII-2.3/
RNAfold	ViennaRNA Web Services	http://rna.tbi.univie.ac.at/cgi-bin/RNAWebSuite/RNAfold.cgi
GenomicRanges v1.56.2	Lawrence et al.^72^	https://github.com/Bioconductor/GenomicRanges
biovizBase v2.64.0	Yin et al.^73^	https://github.com/jorainer/biovizBase
ggplot2 v3.4.2	Wickham et al.^74^	https://github.com/tidyverse/ggplot2
RAWGraphs 2.0	Mauri et al.^75^	https://app.rawgraphs.io/
SRPlots	Tang et al.^76^	http://www.bioinformatics.com.cn/srplot
Inkscape v1.4	NA	https://inkscape.org/
R 4.4.1	NA	https://www.r-project.org/

### Experimental model and study participant details

#### Participant demographics and sample collection

The present investigation includes 112 human blood samples collected from the NS1 antigen test positive Dengue patients, hospitalized at the MAX Super Specialty Healthcare Hospital, New Delhi, India between August and November 2022, which coincided with the peak Dengue infection season in New Delhi, India. Following the acquisition of written consent from the participants, the paramedical team at the MAX Super Specialty Healthcare Hospital, New Delhi, India, gathered 2-3 ml blood samples during the initial hospital visit using the EDTA-coated vials, adhering to the principles outlined in the Declaration of Helsinki. The patients were classified into serotypes –DENV1, DENV2, DENV3- based on the classification of predominant DENV serotype identified from whole transcriptome data (as described in method details). Samples with viral reads at > 50X depth and > 99% coverage were selected for further analysis. Of the 112 samples 58 samples matched the cutoff. Within these 58 samples, the dataset was matched for age and gender. The study was designed in accordance with the declaration of Helsinki and was approved by the institutional ethics committee of CSIR-Institute of Genomics and Integrative Biology, Delhi, India (Ref No: CSIR-IGIB/IHEC/2020-21/01). The patients/participants provided their written informed consent prior to participation in this study.

### Method details

#### Nucleic acid isolation

RNA was extracted from the blood samples using the Qiagen QIAamp RNA Blood Mini Kit, following the manufacturer’s protocol with specific modifications. The incubation and centrifugation times during the erythrocyte lysis step were reduced to 5 minutes. To enhance RNA purification, a 2–3-minute incubation was added during all the washing steps. RNA purity was assessed using a NanoDrop Microvolume Spectrophotometer and further confirmed by running the sample on an agarose gel. The RNA was then stored at -80°C until ready for RNA-Seq library preparation and sequencing.

#### Library preparation and sequencing

Libraries were prepared using 250 ng of RNA with the Illumina TruSeq Stranded Total RNA Library Prep Globin (Illumina, Cat. No. 20020612). The initial steps involved the depletion of globin mRNA and both cytoplasmic and mitochondrial ribosomal RNA, as these are highly abundant in the whole blood. Cleaved RNA fragments were subjected to first-strand cDNA synthesis using reverse transcriptase and random primers. Double-stranded cDNA was then synthesized using DNA polymerase 1 and RNase H, followed by purification with the AMPure XP (Beckman Coulter, A63881). The 3ʹ blunt end of the double-stranded cDNA was adenylated, and sequencing libraries were uniquely indexed and enriched through PCR amplification.

Library quality was assessed using size analysis on an Agilent 2100 Bioanalyzer, and library concentrations were quantified with the Qubit double-stranded DNA (dsDNA) high-sensitivity (HS) assay kit (Thermo Fisher Scientific; catalog no. Q32854). The libraries were then diluted to 4nM and pooled equimolarly with 24 samples per pool. Paired-end sequencing with 2 × 151 read lengths was performed on the NextSeq 2000 platform (Illumina) at a final loading concentration of 650 pM.

#### Sequence alignment to viral genome

Raw sequencing reads obtained from the RNA-seq libraries were quality-checked using FastQC to ensure high data quality (phred score >30) (Figure 5).^57^ Adapter sequences and low-quality reads were trimmed using Trimmomatic.^58^ The processed reads were aligned to the human reference genome (GRCh38.p) using the STAR aligner, with default parameters. The unmapped reads from the human genome alignment were extracted using samtools view and then aligned to the reference genomes of all four dengue virus serotypes (DENV-1, DENV-2, DENV-3, and DENV-4) using STAR aligner.^60^ From the BAM file of mapped reads, variants in the dengue genome were called using bcftools.^61^ The vcf file was generated using bcftools mpileup. To generate the consensus sequence, the VCF file was processed using seqtk, incorporating the detected variants to create a representative viral sequence for each sample (https://github.com/lh3/seqtk). Samples with viral reads at > 50X depth and >99% coverage were selected for further analysis. Of the 112 samples 58 samples matched the cutoff.

The resulting consensus sequences were uploaded to Nextclade (https://clades.nextstrain.org/), a web-based tool for identifying the serotype of dengue viruses and assessing sequence quality. This classification helped categorise the viral sequences into the appropriate serotypes – DENV-1 (n=5) , DENV-2 (n=43), or DENV-3 (n=11) (Tables S1 and S2).

For phylogenetic analysis, it was performed using NGphylogeny.fr site.^62^ For multiple alignment, the MAFFT algorithm was used.^63^ The aligned sequences were used to construct a phylogenetic tree using the FastME method.^64^ The resulting phylogenetic tree was visualised using iTOL (Interactive Tree Of Life), an online tool that allows for the customization and annotation of phylogenetic trees.^65^

#### Nucleotide composition and dinucleotide analysis

##### AT/GC composition analysis

The consensus sequences of each dengue serotype (DENV-1, DENV-2, and DENV-3) were used for calculating nucleotide composition in the distinct genomic regions, including the 5′ untranslated region (5′ UTR), the coding sequence (CDS), and the 3′ untranslated region (3′ UTR). An in-house R script was employed to compute the percentage of adenine-thymine (AT) and guanine-cytosine (GC) content for each region, allowing insights into the overall base composition and its potential influence on genome stability and functionality.

For the CDS, nucleotide composition was further analysed at each codon position (first, second, and third) to identify patterns indicative of codon bias or translational efficiency.

#### Dinucleotide composition and odds ratio calculation

The dinucleotide composition was calculated for the 5′ UTR, CDS, and 3′ UTR regions for each dengue virus serotype. The observed and expected dinucleotide frequencies were determined, where the observed frequency refers to the actual count of a given dinucleotide. Expected frequency was calculated assuming independence between nucleotide pairs, derived from the product of individual nucleotide frequencies.

The odds ratio (OR) for each dinucleotide was computed using the formula:

Odds Ratio = Observed Frequency/Expected Frequency

This metric identified overrepresented or underrepresented dinucleotides in the viral genome: Overrepresented dinucleotides were defined as those with an odds ratio > 1.25. Underrepresented dinucleotides were defined as those with an odds ratio < 0.78.^77^

These analyses provided insights into potential selective pressures and genome evolution, as well as the role of nucleotide and dinucleotide composition in host-virus interactions and immune evasion.

#### Relative synonymous codon usage

The Kazusa Codon Usage Database was utilized to calculate the Relative Synonymous Codon Usage (RSCU) values for the coding sequences (CDS) of DENV-1, DENV-2, and DENV-3 serotypes, as well as for the human host.^66^^,^^78^ The RSCU metric provides a standardized measure of codon usage bias by normalizing codon frequency against the expected usage under uniform synonymous codon choice.

RSCU *i* = Expected frequency of codon *i/*Observed frequency of codon *i*

The RSCU values were interpreted as follows:

1. Preferred codons: RSCU > 1.6, indicating these codons are used more frequently than expected.
2. Non-preferential codons: RSCU < 0.6, indicating these codons are used less frequently than expected.
3. Unbiased codons: RSCU values between 0.6 and 1.6, suggesting a random or balanced usage.

By comparing RSCU values of the DENV serotypes with those of the human host, it was possible to identify codons and associated amino acids that were over- or underrepresented in the viral genome. These findings provide insights into the viral adaptation to the host translational machinery and the selective pressures shaping codon usage. Importantly, RSCU is independent of sequence length, allowing for unbiased comparisons across genomes of varying sizes.

#### Determination of codon usage indices

The vhcub R package was utilized to calculate codon usage bias indices using the consensus sequences generated for each dengue serotype.^67^ This analysis provided insights into the evolutionary and translational dynamics of the dengue virus. The key indices computed were as follows:

1. *Similarity Index:* This metric evaluates the similarity between the codon usage patterns of the dengue virus and its human host. A high similarity index suggests adaptation of viral codon usage to match the host’s translational machinery, potentially enhancing viral protein synthesis efficiency.^79^
2. *Effective Number of Codons (ENc):* The ENc value quantifies the extent of codon usage bias, ranging from 20 (extreme bias with only one codon used per amino acid) to 61 (no bias with all synonymous codons used equally). This measure provides an overall estimate of codon preference across the dengue virus genome.^80^
3. *Codon Adaptation Index (CAI):* The CAI evaluates how well the codon usage of the dengue genome matches the optimal codons of the host. Higher CAI values indicate better adaptation, suggesting potential optimization for translational efficiency and dengue viral fitness in the human host.^81^

These analyses were performed on the DENV 1, 2 and 3 serotypes to compare serotype-specific patterns of codon usage. The results contribute to understanding how the dengue virus optimizes its codon usage for survival and replication in the human host environment.

#### Correspondence analysis

Correspondence Analysis (CA) was performed to compare codon usage patterns across the DENV serotypes 1, 2, 3, and the human host (*H. sapiens*) using RSCU (Relative Synonymous Codon Usage) values. The analysis, conducted with the FactoMineR R package, aimed to identify variations in codon preferences and potential viral adaptations to the host’s translational machinery.^68^ The results were visualized using the fviz_ca_biplot function from the factoextra R package, producing a PCA-like biplot that highlights clustering and divergence in codon usage patterns between the dengue viral serotypes and the human host.^69^ This visualization provides insights into the evolutionary strategies employed by the virus to optimize translation efficiency within the host environment.

#### Open reading frame (ORF) prediction

##### Ribo-seq data collection

The Ribo-seq paired RNA-seq data was downloaded from the Gene Expression Omnibus (GEO) accession number GSE69602 using the sratoolkit (https://github.com/ncbi/sra-tools). Ribo-seq data was generated from the DENV-2-infected Huh-7 cells cultured in the Dulbecco’s modified Eagle’s medium with 10% fetal bovine serum and antibiotics at 37°C in a 5% CO_2_ incubator. Cells were infected with the DENV-2-NGC strain at an MOI of 10 for 1 hour, washed with PBS, and replaced with a pre-warmed medium. For cell fractionation, cycloheximide-treated cells were separated into cytosolic and ER fractions using digitonin and specific buffers. Ribosomal profiling and RNA-seq were performed on cell lysates, with ribosomes isolated by ultracentrifugation, followed by phenol-chloroform extraction and cDNA library preparation. RNA-seq involved rRNA depletion using RiboZero, RNA fragmentation, and cDNA library preparation, which were sequenced on an Illumina HiSeq 2500 platform. Detailed protocols are available in Reid et al., 2018.^33^

##### Ribo-seq and RNA-seq data analysis

Only the ER_infection samples for 12, 24 and 40 hrs were analysed. The raw reads were quality-checked using FastQC, and adapter sequences were removed with Trim Galore (https://github.com/FelixKrueger/TrimGalore). Filtered reads were aligned to the DENV-2 genome (M29095.1) using STAR. The reads stats are provided as (Table S2). The coverage across the genome was also determined using bedtools (Figure S7). The BAM files were analysed with Ribotricer to identify translating ORFs.^70^ The `prepare_orfs’ function was employed to detect ORFs longer than 30 amino acids. Since the phase score cutoff for the DENV-2 was unknown, the `learning_cutoff` function was used to determine the phase score, yielding a mean of 0.055. Potential iORFs from Ribotricer output were filtered based on the read density, minimum read count thresholds, and minimum valid codon fractions. ORFs with >5000 reads, a read density >100, and a valid codon fraction >0.9 were classified as translating iORFs. Two modifications in the ribotricer tool were made, firstly internal ORFs are by default not reported which was modified to report all potential ORFs, secondly, the minimum reads required to predict periodicity was set to 10,000 for this analysis.

##### Filtration of iORFs

Additionally, the DENV-2 consensus FASTA generated from the clinical samples was analyzed using the NCBI ORFfinder to identify potential reading frames, which identified 74 ORFs in the three reading frames and +/- strand (Table S3A). Only the positive strand ORFs with >30 amino acid lengths and reads density across the ORF >100 were considered (Table S3B). Finally, overlaps between both the Ribo-seq/RNA-seq samples and from clinical samples were used to further filter potential iORFs (Table S3C). The denoised profile of read counts for each ORF generated by ribotricer was visualised using the `plotting_ribotricer_profile’ ribotricer tool.

##### Features of iORFs

The filtered viral iORFs from the ribotricer tool were further checked for their possible functions. Taking the protein sequences of these iORFs, the frequency of amino acid between the iORFs and primary/canonical ORF (ORF1) was computed using the ExPASy ProtParam tool.^82^ The amino-acid composition was compared between ORF1 and iORFs using the Mann Whitney test in GraphPad prism. Next, transmembrane helix prediction was performed using the MEMSAT SVM tool using the protein sequences of the iORFs.^83^ It employs a Support Vector Machine (SVM)-based approach to identify the locations, orientations, and functional characteristics of transmembrane regions in proteins. Following this, antigenicity of these iORFs was predicted by computing the number of possible putative peptides that efficiently bind MHC-I and MHC-II molecules using netMHC 4.0 and netMHC-II 2.3 web-based tools (Table S4).^84^^,^^85^ The recommended length of peptide (9 aa) was used for the predictions, on the set of iORFs and ORF1. Lastly, the secondary structure of iORFs was predicted using the RNAfold tool using the default parameters.

### Quantification and statistical analysis

Wherever appropriate and required, categorical data comparisons were conducted through Chi-square testing. All statistical analyses were carried out using a licensed version of GraphPad Prism. For data visualisations, a combination of ggbio,^86^ GenomicRanges,^72^ biovizBase,^73^ ggplot2,^87^ ggbreak,^88^ rawgraphs,^75^ SRPlots,^76^ and Microsoft Excel was employed. Figures were subsequently modified using Inkscape (https://inkscape.org). A significance threshold of *P-value*< 0.05 was applied, unless otherwise specified.

## Supplemental information

Document S1. Figures S1–S7 and Tables S1 and S2

Table S3. Identification and analysis of ORFs in DENV-2 from clinical samples, related to Figure 3 and corresponding evidence from independent Riboseq datasets

Table S4. Analysis of high binding sites in predicted small internal ORFs, related to Figure 4
