# Supplementary material for: Suboptimal dengue genome leverages non-canonical translation mechanisms
Source: iScience. 2025 Apr 15;28(5):112428. doi: 10.1016/j.isci.2025.112428 (PMC12245444; doi:10.1016/j.isci.2025.112428)
Supplement: Document S1. Figures S1–S7 and Tables S1 and S2 [file mmc1.pdf]

iScience, Volume *28*

Supplemental information

Suboptimal dengue genome leverages non-canonical translation mechanisms

Priyanka Mehta, Priti Devi, Sandeep Budhiraja, Bansidhar Tarai, and Rajesh Pandey

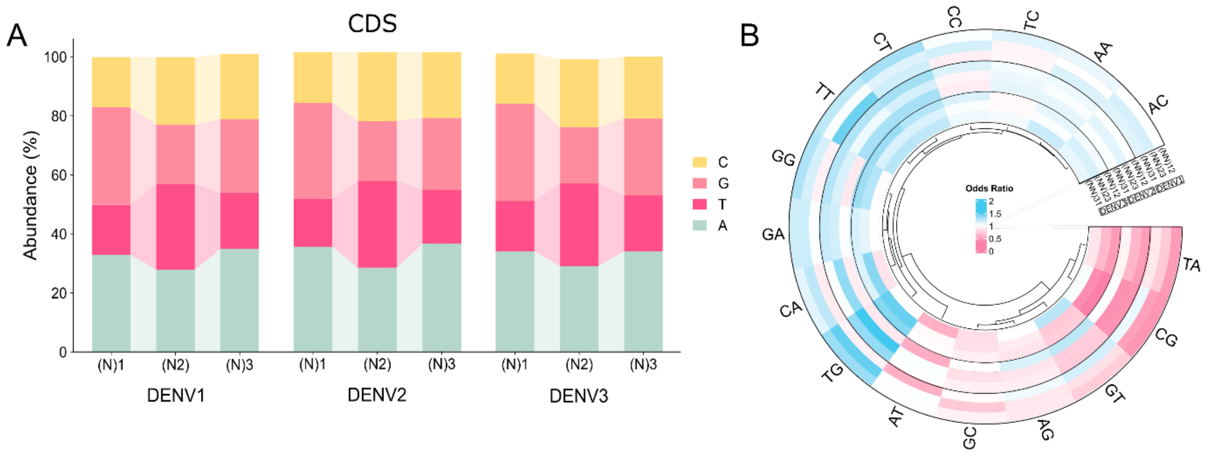

Supplementary Figure S1: Comparison of nucleotide composition at codon positions across DENV serotypes. **(A)** Nucleotide composition at codon positions (N)1, (N)2 and (N)3 across the DENV-1, DENV-2 and DENV-3 serotypes. (**B**) Dinucleotide composition at the codon positions (NN)12, (NN)23, and (NN)31 across the DENV-1, DENV-2, and DENV-3 serotypes.

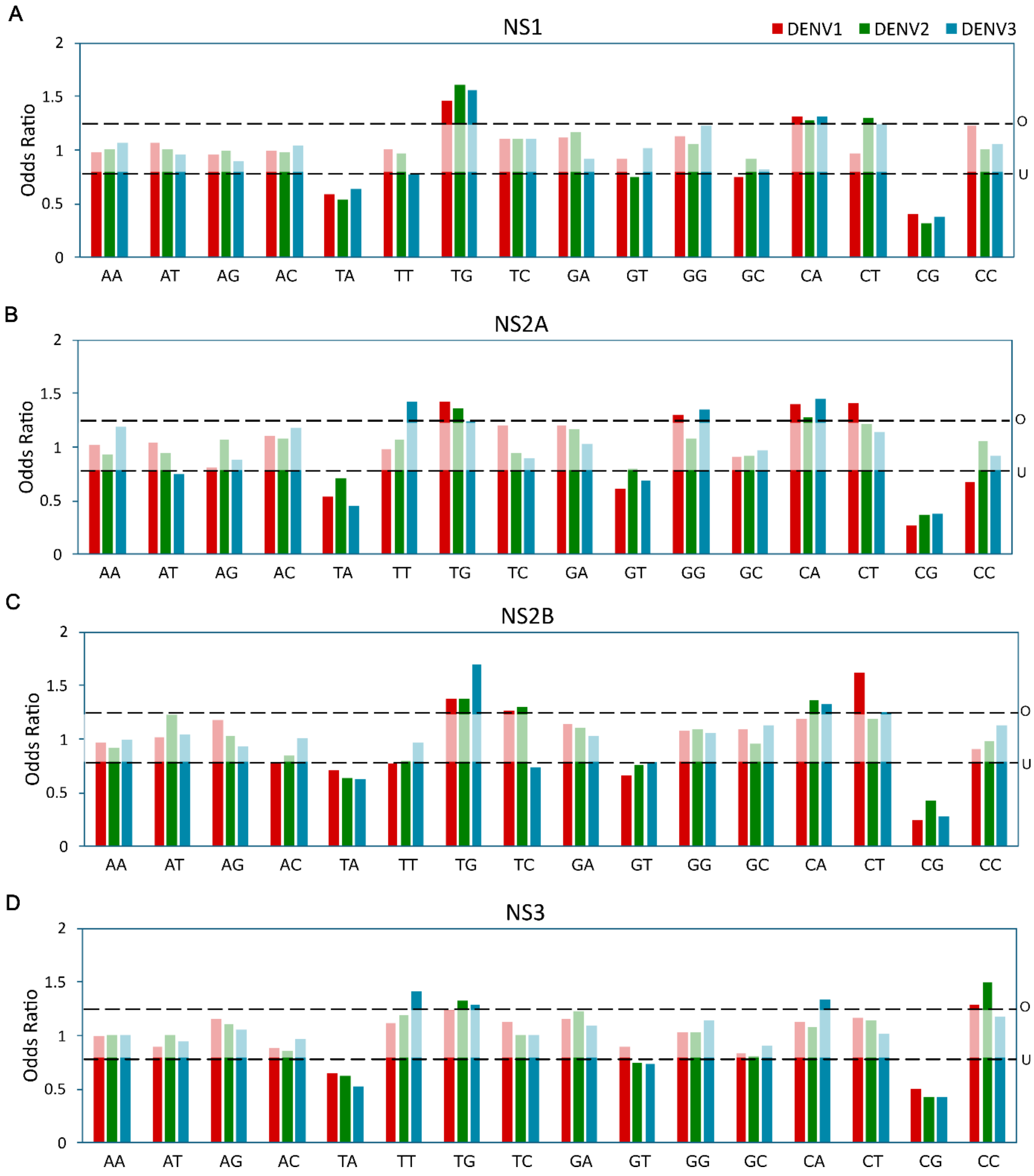

Supplementary Figure S2: Dinucleotide composition across the DENV non-structural genes. (**A**) NS1, (**B**) NS2A, (**C**) NS2B, (**D**) NS3, (**E**) NS4A, (**F**) NS4B, and (**G**) NS5.

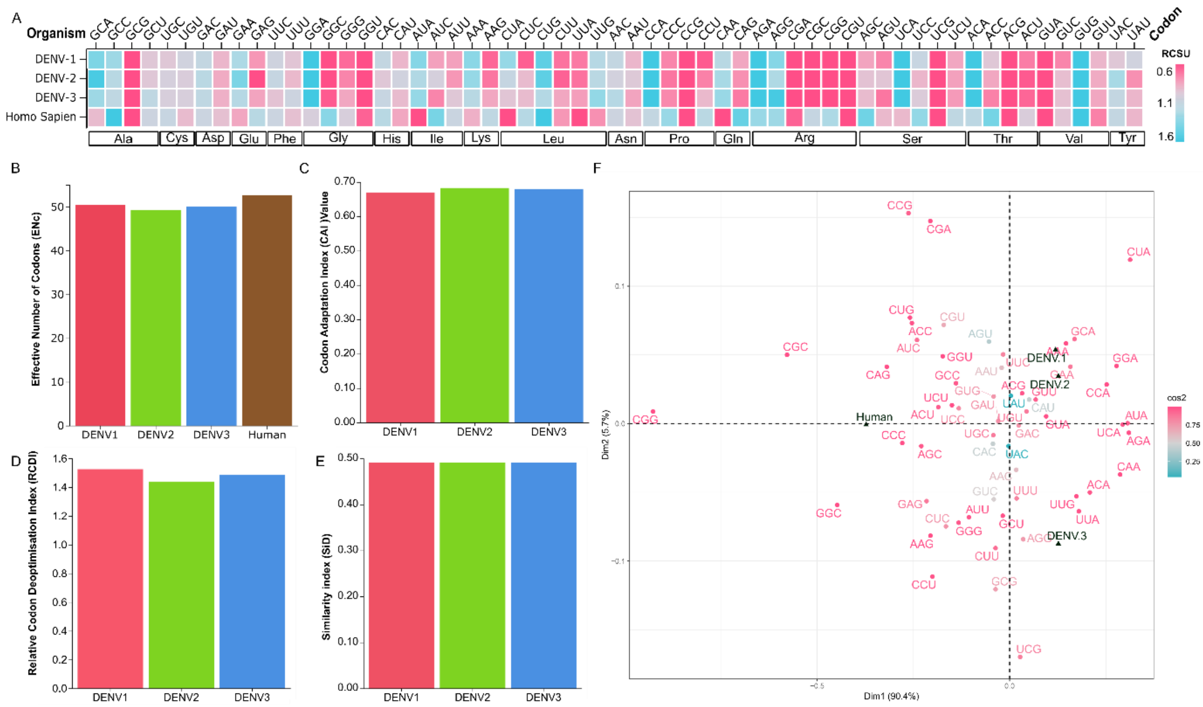

Supplementary Figure S3: Comparative Analysis of Codon Usage Patterns across DENV serotypes and human. (**A**) Heatmap illustrating the Relative Synonymous Codon Usage (RSCU) patterns across the DENV serotypes 1, 2, and 3, and humans. **(B-E)** Bar plots showing comparative indices between the DENV serotypes and humans: (**B**) ENC (Effective Number of Codons) indicating codon usage bias. (**C**) CAI (Codon Adaptation Index) measuring codon optimization. (**D**) RCDI (Relative Codon Deoptimization Index) comparing the codon usage against the host. (**E**) SiD (Similarity Index) representing codon similarity. (**F**) PCA plot displaying codon distribution across first two axes based on correspondence analysis of RSCU values for the CDS regions of the DENV serotypes 1, 2, and 3, and the human host.

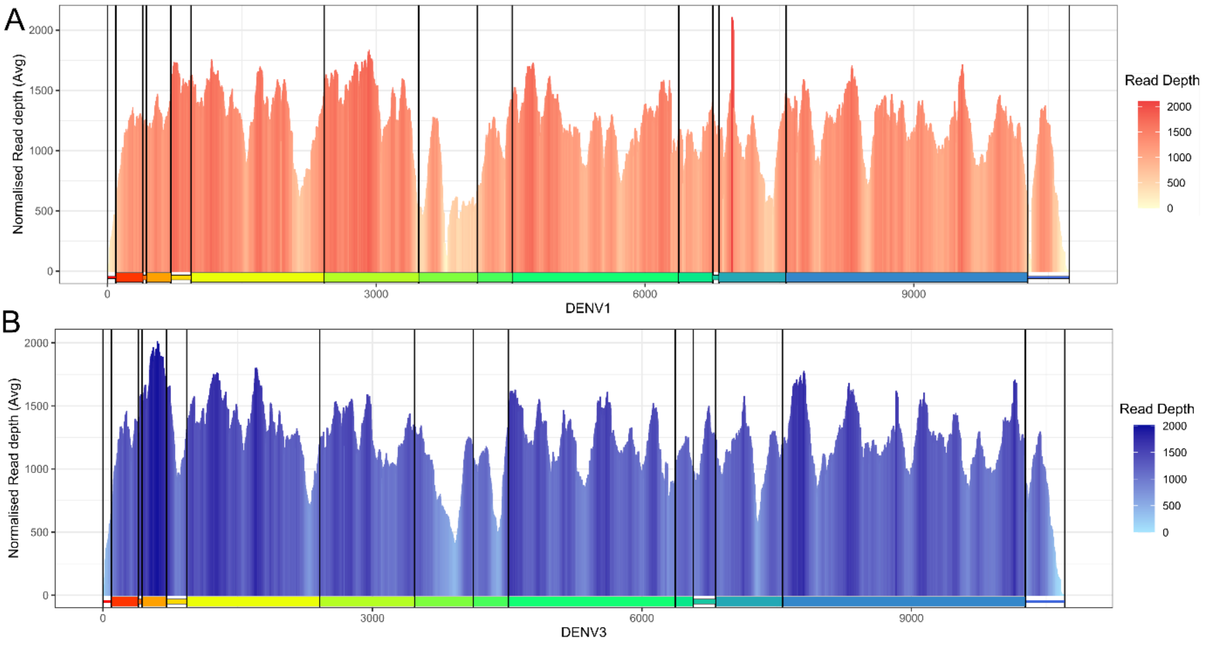

Supplementary Figure S4: Coverage distribution across the genome for (**A**) DENV-1, and (**B**) DENV-3 serotypes derived from the clinical RNA-seq samples.

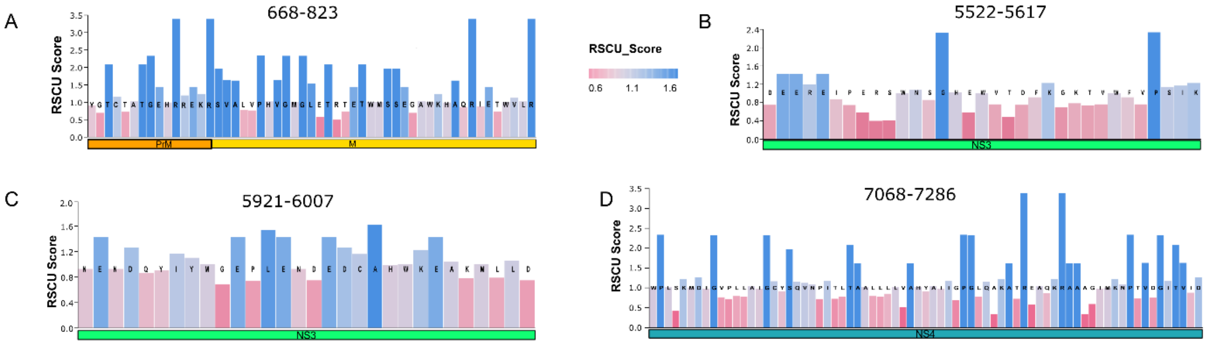

Supplementary Figure S5: The bar plots depict codon usage patterns as RSCU values within the selected iORF genomic regions: **(A)** 668-823 (PrM-M), **(B)** 5522-5617 (NS3), **(C)** 5921-6007 (NS3), and **(D)** 7068-7286 (NS4).

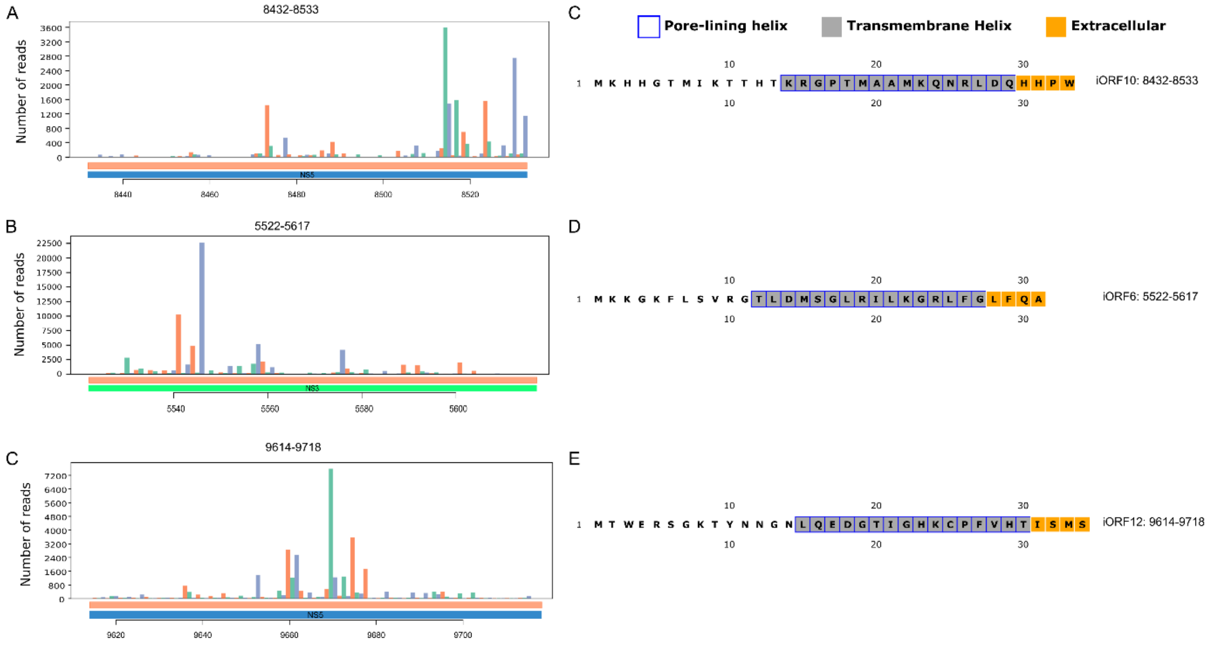

Supplementary Figure S6: Analysis of Potential iORFs and Transmembrane Helix Predictions **(A-C)** Bar plots display ribosomal read profiles predicted for potential iORFs in frame 2 using the Ribotricer tool, with bars representing the position of ribosomal reads in frame 1 (red), 2 (green), and 3 (blue) across the ORFs. (**D-F**) Transmembrane helix prediction across the iORFs , the sequences colored in yellow represent extracellular regions, regions in grey represent transmembrane helix, regions in white are cytoplasmic, while regions with blue border are predicted to form pore-lining helix.

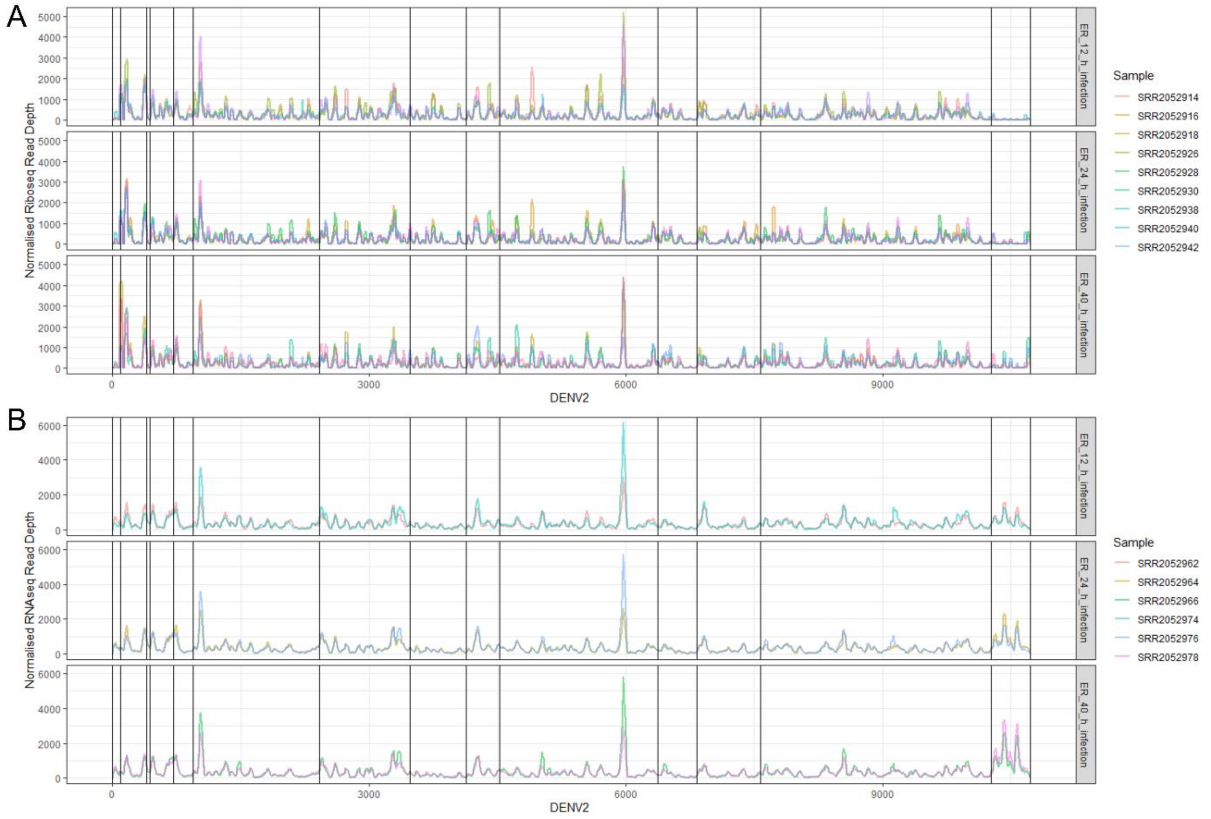

Supplementary Figure S7: Coverage distribution across the genome for **(A)** Dengue Ribo-seq samples, and **(B)** Paired RNA-seq samples distributed across different samples and time-points.

Supplementary Table S1: Statistics for DENV Read Count, Coverage Depth in Clinical Samples and RNA quality score, related to Figure 2a**.**

sample ID	Depth	read count	DENV serotype	Coverage	accession	260/280
IGIB113002314270	67.6635	5467	DENV3	99.66377136	SRR27828895	1.9
IGIB113002156413	61.9087	6619	DENV2	99.4404551	SRR27828898	1.91
IGIB1130388376	259.874	25771	DENV2	99.56168983	SRR27828900	1.97
IGIB1130143348	1071.48	104039	DENV2	99.6922503	SRR27828901	1.87
IGIB1130141653	9749.17	758885	DENV3	99.93462221	SRR27828902	1.89
IGIB113003405741	1535.26	150349	DENV2	99.7109018	SRR27828903	1.93
IGIB113002390340	5886.8	559456	DENV2	99.86943952	SRR27828904	1.89
IGIB113002385876	197.34	15754	DENV3	99.75716821	SRR27828905	1.97
IGIB113002385384	128.578	12319	DENV2	99.68292455	SRR27828906	1.94
IGIB1130417931	68.378	5468	DENV3	99.56103484	SRR27828907	1.91
IGIB113002313519	753.047	59294	DENV1	99.83232417	SRR27828910	1.82
IGIB113002299733	96.9403	9506	DENV2	99.56168983	SRR27828911	1.81
IGIB113002297804	616.86	59176	DENV2	99.65494731	SRR27828912	1.95
IGIB113002190929	2001.4	193320	DENV2	99.85078803	SRR27828913	1.88
IGIB113002393520	602.001	57219	DENV2	99.54303833	SRR27828914	1.83
IGIB113002393366	74.9804	7471	DENV2	99.48708384	SRR27828915	1.83
IGIB113002307585	324.423	31302	DENV2	99.58966707	SRR27828916	2.01
IGIB1130929243	102.936	10603	DENV2	99.44978084	SRR27828917	1.91
IGIB1130922137	718.665	70996	DENV2	99.6922503	SRR27828923	1.86
IGIB113003404387	213.171	17493	DENV1	99.63670238	SRR27828924	1.9
IGIB113002385680	145.413	14903	DENV2	99.64562156	SRR27828925	1.97
IGIB113002376445	3451.83	271617	DENV3	99.74782852	SRR27828926	1.98
IGIB113002224539	581.594	43988	DENV3	99.93462221	SRR27828927	1.83
IGIB113002365687	259.295	21216	DENV1	99.93479273	SRR27828930	1.93
IGIB1130532683	150.015	14455	DENV2	99.81348503	SRR27828934	1.91
IGIB1130486503	10234.6	801031	DENV3	99.72914915	SRR27828936	1.84
IGIB1130492220	535.001	50964	DENV2	99.58966707	SRR27828937	1.98
IGIB1130427008	8616.66	847285	DENV2	99.7295533	SRR27828939	1.86
IGIB113002300283	359.696	35628	DENV2	99.54303833	SRR27828942	2.02
IGIB1130581789	388.018	39012	DENV2	99.57101557	SRR27828945	1.86
IGIB1130492817	516.518	51895	DENV2	99.58034132	SRR27828946	1.87
IGIB1130496737	893.365	86659	DENV2	99.62697006	SRR27828950	1.91
IGIB1130493597	158.529	15919	DENV2	99.90674252	SRR27828951	1.86
IGIB1130488088	613.662	48495	DENV3	99.83188568	SRR27828954	1.91
IGIB1130487048	137.734	13614	DENV2	99.44978084	SRR27828955	1.93
IGIB113001664199	756.161	59348	DENV3	99.67311105	SRR27828959	1.82
IGIB1130928465	1629.66	128880	DENV1	100	SRR27828960	1.95
IGIB1130924841	98.7666	10557	DENV2	99.6922503	SRR27828961	1.89
IGIB1130923527	218.584	21265	DENV2	99.84146228	SRR27828962	1.92
IGIB113002372457	75.7829	6301	DENV1	99.68327899	SRR27828965	1.91
IGIB113002358285	125.247	12888	DENV2	99.6922503	SRR27828967	1.95
IGIB1130349380	140.523	13597	DENV2	99.4404551	SRR27828974	1.93
IGIB1130259880	1357.37	130944	DENV2	99.73887905	SRR27828976	1.86
IGIB113002298284	59.0692	5891	DENV2	99.64562156	SRR27828977	1.89

IGIB113002257842	2916.95	276442	DENV2	99.88809102	SRR27828980	1.42
IGIB1130483166	134.494	10558	DENV3	99.81320631	SRR27828981	1.85
IGIB1130395724	369.931	35804	DENV2	99.54303833	SRR27828983	1.9
IGIB1130170839	200.027	19058	DENV2	99.45910659	SRR27828985	1.88
IGIB1130151498	107.256	8429	DENV3	99.56103484	SRR27828986	1.9
IGIB113000849750	1898.79	190010	DENV2	99.93471976	SRR27828989	1.94
IGIB113083612	53.4539	5683	DENV2	99.48708384	SRR27828991	1.86
IGIB1130661232	85.2785	8419	DENV2	99.12337965	SRR27828993	2.03
IGIB1130631658	511.473	49879	DENV2	99.55236408	SRR27828994	1.84
IGIB1130567172	66.6015	7148	DENV2	99.56168983	SRR27828995	1.89
IGIB113078087	103.232	10261	DENV2	99.25394013	SRR27828997	1.89
IGIB1130757484	826.002	81490	DENV2	99.74820479	SRR27828998	1.92
IGIB1130658717	97.137	9710	DENV2	99.48708384	SRR27829000	1.94
IGIB113065285	534.721	52395	DENV2	99.68292455	SRR27829002	1.93
IGIB113002348939	124.978	13021	DENV2	99.54303833	SRR27829004	1.95

Supplementary Table S2: Statistics for DENV Read Count and Coverage Depth from Riboseq and RNAseq Samples, related to figure 2c

sample ID	type	sample_description	DENV serotype	Depth	read_counts
SRR2052914	riboseq	ER_12_h_infection	DENV2 (M29095.1)	1139.22	419721
SRR2052916	riboseq	ER_24_h_infection	DENV2 (M29095.1)	2331.15	891384
SRR2052918	riboseq	ER_40_h_infection	DENV2 (M29095.1)	8977.13	3297990
SRR2052926	riboseq	ER_12_h_infection	DENV2 (M29095.1)	379.319	136731
SRR2052928	riboseq	ER_24_h_infection	DENV2 (M29095.1)	1399.71	579300
SRR2052930	riboseq	ER_40_h_infection	DENV2 (M29095.1)	1827.21	688544
SRR2052938	riboseq	ER_12_h_infection	DENV2 (M29095.1)	72.4673	33343
SRR2052940	riboseq	ER_24_h_infection	DENV2 (M29095.1)	248.491	108611
SRR2052942	riboseq	ER_40_h_infection	DENV2 (M29095.1)	254.472	99708
SRR2052962	rnaseq	ER_12_h_infection	DENV2 (M29095.1)	36.3028	23650
SRR2052964	rnaseq	ER_24_h_infection	DENV2 (M29095.1)	302.737	125928
SRR2052966	rnaseq	ER_40_h_infection	DENV2 (M29095.1)	1361.36	483570
SRR2052974	rnaseq	ER_12_h_infection	DENV2 (M29095.1)	113.117	50000
SRR2052976	rnaseq	ER_24_h_infection	DENV2 (M29095.1)	1158.73	423557
SRR2052978	rnaseq	ER_40_h_infection	DENV2 (M29095.1)	1034.45	383269
